# Size-dependent aggression towards kin in a cannibalistic species

**DOI:** 10.1093/beheco/arac020

**Published:** 2022-03-25

**Authors:** Chloe A Fouilloux, Lutz Fromhage, Janne K Valkonen, Bibiana Rojas

**Affiliations:** 1 Department of Biology and Environmental Science, University of Jyvaskyla, P.O. Box 35, 40014 Jyväskylä, Finland; 2 Department of Interdisciplinary Life Sciences, Konrad Lorenz Institute of Ethology, University of Veterinary Medicine Vienna, Savoyenstraße 1, 1160 Vienna, Austria

**Keywords:** cannibalism, kin discrimination, larval aggression, poison frog, tadpole

## Abstract

In juveniles extreme intraspecies aggression can seem counter-intuitive, as it might endanger their developmental goal of surviving until reproductive stage. Ultimately, aggression can be vital for survival, although the factors (e.g., genetic or environmental) leading to the expression and intensity of this behavior vary across taxa. Attacking (and sometimes killing) related individuals may reduce inclusive fitness; as a solution to this problem, some species exhibit kin discrimination and preferentially attack unrelated individuals. Here, we used both experimental and modeling approaches to consider how physical traits (e.g., size in relation to opponent) and genetic relatedness mediate aggression in dyads of cannibalistic *Dendrobates tinctorius* tadpoles. We paired full-sibling, half-sibling, and non-sibling tadpoles of different sizes together in an arena and recorded their aggression and activity. We found that the interaction between relative size and relatedness predicts aggressive behavior: large individuals in non-sibling dyads are significantly more aggressive than large individuals in sibling dyads. Unexpectedly, although siblings tended to attack less overall, in size-mismatched pairs they attacked faster than in non-sibling treatments. Using a theoretical model to complement these empirical findings, we propose that larval aggression reflects a balance between relatedness and size where individuals trade-off their own fitness with that of their relatives.

## Teaser

Before you eat someone, you have to attack them first. Here, we investigated the factors that shape aggression in the cannibalistic tadpoles of the dyeing poison frog. We find that aggression depends on both size and relatedness: when set in pairs, large tadpoles are half as aggressive towards their smaller siblings than to nonsibs. It looks like belonging to the same family provides some protection against aggression, though no one is ever truly safe.

## INTRODUCTION

Aggression is often a necessary precursor to cannibalism, as individuals must subdue their counterpart before consuming them (Sakakura and Tsukamoto 1997; Caldwell and de Araujo 1998; Lund et al. 2016). In juveniles, which typically do not hold territories nor compete for mates, the function of escalated aggression is primarily to monopolize nutritional resources (either realized or potential) as most of their energy is invested into growth. In systems with sibling aggression, fighting represents an important potential advantage in early development for securing resources (Drummond et al. 2003; Naidenko and Antonevich 2009); in cannibalistic species, the factors that shape opponent assessment are vital, as there is the threat of interactions escalating to death. Thus, cannibalism is often conditional on the assessment of either the environment (food availability: Mayntz and Toft 2006; Dugas et al. 2016a, conspecific density: Maret and Collins 1994, Frankino and Pfennig 2001; or a combination of the two: Wildy et al. 2001) or the opponent (size and relatedness: Dugas et al. 2016b, condition: Ibáñez and Keyl 2010).

Empirically, many studies have found that winners of cannibalistic interactions are larger than losers (Claessen et al. 2004; Ibáñez and Keyl 2010; Barkae et al. 2014; Rojas 2014), although exceptions exist when larger individuals are weakened (Richardson et al. 2010) or when individuals compensate for their size with increased aggressiveness (Issa et al. 1999). Kinship between individuals can also explain aggression. This has been shown to be an important factor in several cannibalistic species that demonstrate kin discrimination and avoid eating kin (Pfennig et al. 1994; Pfennig and Frankino 1997; van den Beuken et al. 2019), although there are also examples of cannibals consuming their kin without avoidance (Boots 2000; Gray et al. 2009). Although differences in opponent size and relatedness have individually been identified as variables that shape cannibalistic decisions, the interaction between these two variables has yielded diverse results across taxa where, for example, studies have reported a strong interactive effect in earwigs (Dobler and Kölliker 2011), the absence of size effect in spiders (Bilde and Lubin 2001; Roberts et al. 2003), and both a stage and phenotype dependent adversity where spadefoot toads are less likely to cannibalize other cannibals (Pfennig 1999) as well as more developed siblings (Dugas et al. 2016b). Ultimately, more work is needed to tease apart the factors influencing decision-making in juvenile cannibals in a broader range of taxa. Notably, in low-fecundity systems where each case of cannibalism may represent a substantial loss to the parents, understanding the adaptive significance of cannibalism seems all the more pressing.

*Dendrobates tinctorius* is a Neotropical poison frog with parental care whose larvae are facultative cannibals (Rojas 2014). Tadpoles are often deposited by their fathers in ephemeral pools of water, where they are confined until metamorphosis (Rojas and Pašukonis 2019). While tadpoles are most often transported singly, the ephemeral pools in which they are deposited can have multiple tadpoles of various developmental stages (Rojas and Pašukonis 2019) and degrees of relatedness (Rojas B, and Ringler E, unpublished data). In these environments, cannibalism is common (Rojas 2014, 2015), but not necessary for the successful development and metamorphosis of an individual tadpole. In closely related poison frogs, cannibalism is usually an outcome of sequentially intensified attacks (Summers and Symula 2001; Gray et al. 2009), although exceptions where tadpole aggression does not include cannibalism exist (i.e., obligate egg-feeders with parental care, Dugas et al. 2016a). Here, we aim to better understand what drives cannibals to express aggression towards conspecifics and disentangle the apparent variation that exists in this behavior.

For *D. tinctorius,* the costs of cannibalism are direct, as attacking kin can reduce inclusive fitness and the potential for injury (even with a small counterpart) is high. The long-term potential benefits, on the other hand, are yet to be established in detail. Fundamental work in systems with sibling aggression posits that aggression towards kin evolves when the benefits are greater than the (in)direct fitness costs associated with fights (Parker et al. 1989). For example, consuming a conspecific could shorten the cannibal’s time to metamorphosis and increase size thereafter (as observed in frogs: Crump 1990; spiders: Mayntz and Toft 2006; salamanders: Wildy et al. 1998). This could, in turn, translate into escaping precarious conditions and improving fitness (Wissinger et al. 2004). Here we build game theory models post hoc to theoretically investigate the evolutionary forces that shape aggressive encounters in *D. tinctorius.* As we will show in the theoretical part of this study, small changes in the assumptions about the size-dependent costs of aggressiveness can lead to qualitatively different predictions of behavior. We therefore derive a range of alternative predictions to serve as alternative hypotheses for the empirical component of our study. In this experiment we considered size and relatedness to better understand the basis of aggression in a cannibalistic species. We conducted behavioral assays between dyads of *D. tinctorius* tadpoles, and measured aggression and activity in response to changes in relative size differences and relatedness between pairs.

In the context of cannibalism, recording general activity levels in addition to aggression itself can help to elucidate the underlying behavioral mechanisms (Kralj-Fišer et al. 2012; Adriaenssens and Johnsson 2013; Vallon et al. 2016); for example, an increase in activity could be a result of either attacking or evasion by tadpoles, whereas a decrease could be either stealth or a freezing response. It is important to note that although aggression has often been used as a proxy for cannibalism throughout this family (Caldwell and De Araujo 1998; Summers and Symula 2001), we cannot fully disentangle whether conspecific aggression is truly an attempt at predation or an act of resource defense, where more aggressive tadpoles would acquire a foraging benefit. Nevertheless, because both tadpoles are confined to the same pool of water throughout development, either predation or resource-holding behaviors converge on the same outcome of additional feeding opportunities. Together, these experiments and models contribute to our understanding of how intraspecies aggression is shaped by the relatedness and size differences of competitors that may cannibalize each other.

## METHODS

### Study species

*Dendrobates tinctorius* has elaborate parental care. Males attend small terrestrial clutches and transport newly hatched tadpoles, one or two at a time, to pools of water where they are left until metamorphosis. Males carrying more than one tadpole at once can be seen either depositing both tadpoles in the same pool or distributing tadpoles between pools (Rojas and Pašukonis 2019). The tadpoles are omnivorous and frequently demonstrate cannibalism (Rojas 2014, 2015); despite this, it is not unusual to see several tadpoles, at various stages of development, coexisting within the same pool in the wild (Rojas and Pašukonis 2019; Fouilloux et al. 2021).

We used tadpoles from a breeding laboratory population of *D. tinctorius* kept at the University of Jyväskylä, Finland. We used a paternal half-sibling design as it could be expected that paternal half-siblings are more likely to co-occur as a result of fathers reusing pools after multiple transport events. Tadpole dyads were assigned in response to 1) individuals needing to be visually distinguishable from each other (i.e., size), and 2) the laboratory mating schedule/network, which was prioritized so as to not stress the animals from overbreeding. Most breeding pairs laid clutches (3–7 eggs) weekly, which allowed us to use tadpoles of diverse sizes throughout the experiment. Adult pairs were each housed in a 115L terrarium that contained layered expanded clay, leaf-litter, moss substrate and were equipped with a shelter, logs, and live plants. Terraria were maintained at 26 °C (±2 °C) and were automatically misted with reverse osmosis water four times a day (maintaining a humidity around 95%) and lit with a 12:12 photoperiod. Frogs were fed live *Drosophila* fruit flies coated in vitamin supplements five times per week. Tadpoles were raised singly in 10 × 6.5 × 5 cm containers that were filled with spring water, and fed ad libitum a diet of fish food (JBL NovoVert flakes) three times a week. Adult and tadpole health and water levels were checked daily.

### Behavioral trials

Pairs of tadpoles of different degrees of relatedness (full-sibling, half-sibling, non-sibling) were placed together in an arena. Tadpoles in early larval development were used, that is, before the toe differentiation in hind legs development to control for possible life-history effects (stage 31, Gosner 1960). Experimental tadpole weight ranged from 0.04 g to 0.38 g, and mass differences between pairs ranged from 0.03 g to 0.30 g. Blinding in the experiment was not possible, as the set-up and experiment were conducted by the same person, but the order of trials was assigned randomly. The arena was an 18.5 cm by 12 cm clear plastic container filled with 400 mL of spring water. Initially, each tadpole was placed on either side of an opaque partition dividing the arena; this partition kept tadpoles separated but allowed water to flow throughout the container. After an acclimation period of one hour, tadpole activity (resting, swimming) of the separated individuals was recorded every 15 s for 10 min.

After the acclimation and separated observation, the barrier was removed and tadpole interactions were recorded for 60 min. Behaviors (resting, swimming, biting, and chasing; see Supp. Table 1 for descriptions) were recorded for both tadpoles every 15 s. Tadpoles were visually distinguishable due to size differences, as heavier tadpoles were larger. Individuals were photographed and weighed before the beginning of each trial to establish initial tadpole condition, and were only used once (n_Trial_ = 15 for each relatedness level, *n *= 90 tadpoles for the entire experiment).

Trials were ended prematurely if tadpoles demonstrated aggression levels that would cause severe damage or death (where bites lasted for more than 2 s, recorded as “potential lethal attack”). Although aggression was common, potential lethal attacks were rare, occurring in only 3/45 trials. There were no tadpole deaths as a result of the behavioral trials, and all tadpoles were kept and reared in the laboratory after the experiment. Assay methods followed the Association for the Study of Animal Behaviour’s guidelines for the treatment of animals in behavioral research and teaching (ASAB 2018), and were done with the approval of the National Animal Experiment Board at the Regional State Administrative Agency for Southern Finland (ESAVI/9114/04.10.07/2014).

### Statistical analysis

All models and statistics were performed in the program R (v. 3.6.1, R Development Core Team 2019) with additional packages “glmmTMB” (Brooks et al. 2017), “coxme” (Therneau 2020), “dplyr” (Wickham et al. 2018), “tidyr” (Wickham et al. 2019). Activity and aggression analyses (see below) took into account pair identity (Pair_ID) and family (breeding pair) level random effects. We included pair ID as a random effect because we needed to consider that individuals were not independent within pairs; similarly for family, the behavior of siblings was likely partly correlated, and we wanted to account for that possibility. Differences in duration of trials during experiments (*n* = 3/45 trials ended early due to potential lethal attacks) were taken into account by offsetting models with a trial duration. The structure of the aggression and activity models was based on the interaction between relative size (two-level categorical, where tadpoles were assigned a relative size (large/small) within a dyad) and relatedness (three-level categorical, sibling/half-sibling/non-sibling). Based on the size effect having predicted cannibalism in previous experiments in the wild (Rojas 2014), which reported the effect of size on the latency to cannibalism, we hypothesized that relative size differences must play a part in shaping aggressive decisions between kin. Residual diagnostics (zero-inflation, residual patterns, and over/underdispersion) were checked using the “DHARMa” (Hartig 2020) package, all of the final models passed diagnostic checks.

### Activity levels

Tadpole activity was categorized as “resting” and “swimming” (see Supp. Table 1 for details). Tadpole activity was observed during post-acclimation (10 min) and experimental (max. 60 min) periods. These measures provided an assessment of how tadpoles behaved before and after visual/physical contact, and help contextualize the role of activity versus aggression. Activity was coded as counts and was modeled in a generalized linear mixed model framework (GLMM). Because these data were overdispersed, they were modeled using a negative binomial parameterization, which adjusts the variance independently of the mean.

### Overall aggression

Aggression between tadpoles was observed as chasing or biting, which were recorded as counts. These two behaviors were combined to represent “total aggression”. These data provide a direct measure of aggression between dyads, which have generally been considered as a precursor to cannibalism in this family. These data were fit with a Poisson family with a log link.

### Latency to first bite

Latency data were built by selecting the “first biter” within a pair, which involved subsetting the original data set. We modeled latency to first bite using a mixed effect Cox proportional hazards model. Survival object was parameterized with respect to latency to first bite event and absolute biting (0/1, where 0 represents no biting occurred during the trial) in response to the interaction of relatedness and mass difference between tadpole dyads. The first bite within a dyad is of interest because taste may play a role in kin recognition (as with salamanders; Pfennig et al. 1994); thus, this behavior could serve as an initial assessment, but is risky as it exposes tadpoles to potential attacks. Mass difference was calculated as the difference between tadpole pairs: this value was always positive because large tadpoles were always heavier. Using subsetted data, each pair identity was independent, so only “Family” was used as a random variable.

### Game theory model

We modeled pairwise interactions between tadpoles arbitrarily labeled as 1 and 2. We assumed that only one tadpole per pair survives (“wins”), and that the probability of winning depends on each individual’s competitive strength. Competitive strength $\theta_{i}$ of tadpole *i* was calculated based on its relative **size**, $s_{i}$ and its **aggressiveness**, $a_{i}$ as $\theta_{i}=s_{i}\cdot a_{i}$. This multiplicative formulation reflects the biological idea that a given increment in aggressiveness should have a greater effect on a large than a small tadpole’s competitive strength. Individual 1’s probability of winning is given by its **relative competitive strength**, as $\omega_{1}=\;\frac{\theta_{1}}{\left(\theta_{1}+\theta_{2}\right)}$. The reproductive success (“**direct fitness**”, $\upsilon_{i}$) of the winning tadpole was modeled under three assumptions: (*1a*) $\upsilon_{i}$ is size-independent, as $\upsilon_{i}=1-{a_{i}}^{2}$; (*1b*) $\upsilon_{i}$ is proportional to size (for a given level of aggressiveness), as $\upsilon_{i}=s_{i}-{a_{i}}^{2}$; and (*1c*) $\upsilon_{i}$ is size-dependent due to aggressiveness being costlier for smaller tadpoles, as $\upsilon_{i}=1-{\left(\frac{a_{i}}{s_{i}}\right)}^{2}$ (see Figure 4 for visualization). In all three formulations costs increased at an accelerating rate, such that low levels of aggression had low costs whereas high levels of aggression could be extremely costly; this was done to account for the increasing danger and energy expense associated with more violent behaviors.

**Figure 1:**
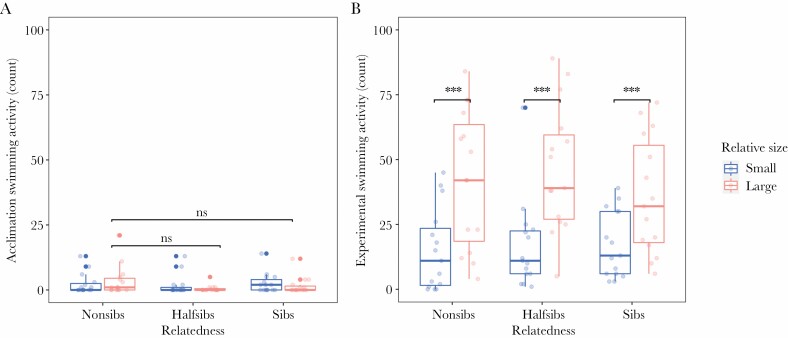
Tadpole activity levels before and during experimental trials. Panel (**A**) shows the post-acclimation activity of tadpoles. We found no difference in swimming between large and small tadpoles or relatedness treatments during this phase. Panel (**B**) shows experimental activity throughout behavioral trials. Large tadpoles were significantly more active than small tadpoles during assays. N_Trial_ = 15 for each relatedness level. Large tadpoles are in pink and small tadpoles in blue. Boxplot medians are depicted by thicker lines, whiskers span ± 1.5 * interquartile range.

**Figure 2:**
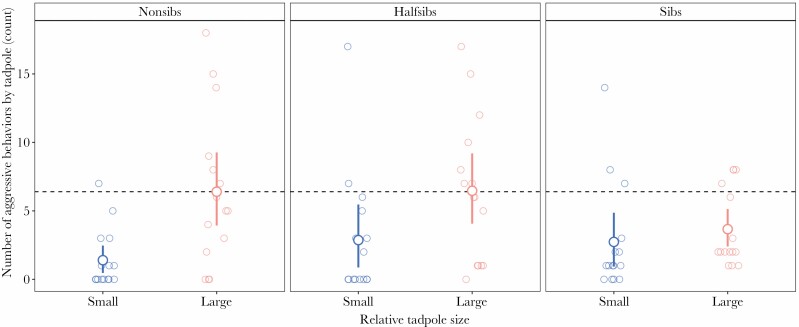
Differences in aggression across relatedness treatments with respect to relative size between dyads. Point ranges indicate mean for each category with bootstrapped 95% confidence intervals. Pink points represent large tadpoles and blue dots represent small tadpoles. Dashed line indicates mean aggression for large tadpoles from non-sibling dyads. N_Trial_ = 15 for each relatedness level. There was significantly less aggression by large tadpoles from siblings dyads when compared with large tadpoles from non-siblings dyads.

**Figure 3:**
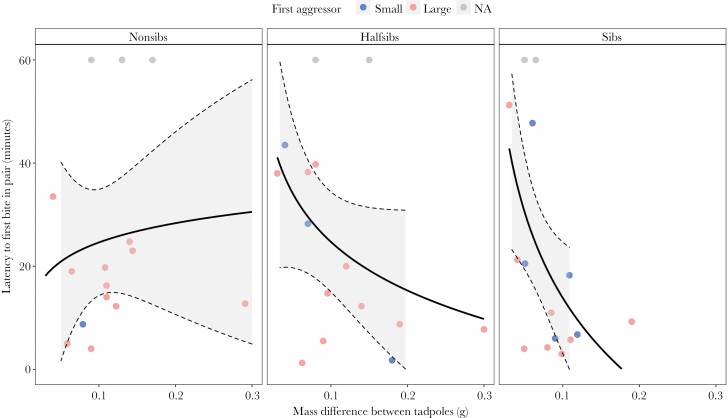
Latency to first bite between tadpole dyads. Points are colored by the first biter’s relative size within dyads. Lines are fit with a GLM smoother with a y ~ log(x) formula and shaded regions represent 95% confidence intervals. There is an inversion in behavior as weight difference between dyads increases, where sibling pairs with large weight differences attacked significantly faster than non-siblings. Dyads where there were no aggressive behaviors were accounted for by assigning them the maximum time limit (60 min). N_Trial_ = 15 for each relatedness level.

**Figure 4:**
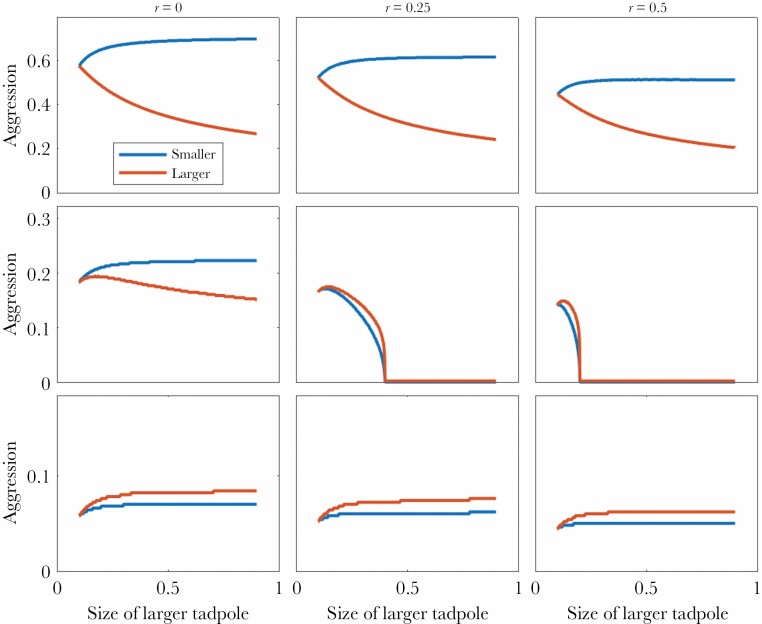
Optimal aggressiveness of dyads of tadpoles as a function of relative size difference for three different levels of relatedness (represented in panel columns) and three sets of assumptions (represented in panel rows). **First row:** direct fitness was assumed to be size-independent. **Second row**: direct fitness was assumed to be proportional to size. **Third row**: aggressiveness was assumed to be costlier for smaller tadpoles. The smaller tadpole’s size was held fixed at s_i_ = 0.1; plotted lines show aggression levels in response to the increasing difference in size between dyads. The larger tadpole’s size is shown on the x-axis.

Finally, the inclusive fitness of the surviving tadpole was calculated as $\upsilon_{1}-r\;\upsilon_{2}$, where *r* is the relatedness between the pair. This formulation reflects the idea that winning involves the killing of a relative that would have had reproductive success $\upsilon_{2}$ had it survived. The inclusive fitness of the losing tadpole is zero, because the losing tadpole neither reproduces nor affects the other tadpole’s reproduction. We calculated the expected (i.e., probability-weighted mean) inclusive fitness of tadpole 1 as *F*_1_ = $\omega_{1}$($\upsilon_{1}-r\;\upsilon_{2})$. For given values of $s_{1}$, $s_{2}$, and $a_{2}$ we numerically determined individual 1’s optimal aggression level as the value of $a_{1}$ that maximizes its expected inclusive fitness. By computing individual 1’s “best response” aggression level for any given $a_{2}$ which its opponent might exhibit, we then identified pairwise optimal aggression levels that are best responses to each other.

## RESULTS

### Activity levels

We observed tadpole activity during both post-acclimation and experimental phases. While tadpoles were separated by an opaque barrier during the post-acclimation phase (but water still freely moved throughout the arena) we did not detect any differences in activity between any of the experimental treatments. During the experiment, however, we found that large tadpoles across all relatedness treatments were significantly more active than small tadpoles (negative binomial GLMM, CI: 1.79–3.58, *z* = 5.23, *P* < 0.001; see Figure 1, Table 1).

**Table 1:** Summary of negative binomial GLMM with linear parameterization of tadpole activity. (A) activity of tadpoles was not affected while tadpoles were physically separated. (B) large tadpoles were overall more active once the barrier was removed. Models for (A) and (B) were predicted by interactive effects of relative size and relatedness. Tadpole dyads (Pair_ID) and family were accounted for as random effects, CI represents 95% confidence interval. Differences in trial time during the experiment (*n* = 3/45) were accounted for by using duration as offset in the model. σ^2^ represents residual variance and τ_00_ represents random intercept variance

(A)	Post-acclimation activity			
Predictors	Estimate	CI	*z*	*P*
(Intercept)	0.72	−0.14–1.58	1.64	0.101
Half-siblings	−0.63	−1.87–0.62	−0.98	0.326
Siblings	0.24	−0.78–1.27	0.47	0.639
Size (large)	0.39	−0.57–1.34	0.79	0.428
Half-siblings: size (large)	−0.63	−2.33–1.07	−0.73	0.466
Siblings: size (large)	−0.90	−2.30–0.51	−1.25	0.211
**Random effects**				
σ ^2^	1.68			
τ _00_ _Pair_ID_	0.19			
τ _00_ _Family_	< 0.001			
(B)	Experimental activity			
Predictors	Estimate	CI	*z*	*P*
(Intercept)	0.00	0.00–0.01	−22.21	**<0.001**
Half-siblings	1.25	0.66–2.36	0.69	0.489
Siblings	1.52	0.82–2.80	1.34	0.181
Size (large)	3.46	1.96–6.09	4.30	**<0.001**
Half-siblings: size (large)	0.81	0.38–1.73	−0.54	0.588
siblings: size (large)	0.51	0.24–1.08	−1.76	0.079
Random effects				
σ ^2^	0.43			
τ _00_ _Pair_ID_	<0.001			
τ _00_ _Family_	0.01			

Bold values represent significant values.

When comparing models, we found that random effects of pair ID had higher between-subject variance (τ _00_ = 0.19) than tadpole family (τ _00_ = <0.001) during post-acclimation activity (Table 1, Panel A), indicating that when separated, there was less variation in behavior on a family level. Yet, while interacting during the experiment this difference disappears (Table 1, Panel B). In both cases, between-subject variance is low, indicating that across families and pairs of tadpoles, activity levels are similar.

### Overall aggression

The total aggression expressed by individuals could be predicted by the interaction between relative size and relatedness between dyads. We found that the interaction term of the model was significant overall (ANOVA, *P* = 0.004, $\chi^{2}$ = 10.905, df = 2). Large tadpoles from sibling dyads were significantly less aggressive than the large tadpoles from non-sibling dyads, exhibiting almost half the amount of aggressive behaviors as large non-siblings (Figure 2, Poisson GLMM, *z* = −3.170, *P* = 0.002, Table 2). Half-siblings were not significantly different from either treatment. After our expectations of creating unique pair interactions, the random effect of pair identity had a high between group-variation (τ _00Pair_ID_ = 1.04, Table 2), but families differed little from each other (τ _00Family_ = 0.13, Table 2).

**Table 2:** Summary of Poisson GLMM of tadpole aggression. Total aggression (total count of biting and chasing) was predicted by the interaction between relative size (two-level categorical variable) and relatedness. Tadpole dyads (Pair_ID) and family were accounted for as random effects, CI represents 95% confidence interval. Differences in trial time during the experiment (*n* = 3/45) were accounted for by using duration as offset in the model. σ^2^ represents residual variance and τ_00_ represents random intercept variance

	Total aggression			
Predictors	Estimate	CI	*z*	*P*
(Intercept)	−8.03	−8.79–−7.28	−20.87	**<0.001**
Half-siblings	0.42	−0.60–1.45	0.81	0.416
Siblings	0.54	−0.43–1.50	1.09	0.275
Size (large)	1.42	0.85–1.98	4.92	**<0.001**
Half-siblings: size (large)	−0.40	−1.21–0.40	−0.98	0.327
Siblings: size (large)	−1.12	−1.82–−0.43	−3.17	**0.002**
Random effects				
σ ^2^	0.34			
τ _00_ _Pair_ID_	1.04			
τ _00_ _Family_	0.13			

Bold values represent significant values.

### Latency to first bite

The initial aggression between tadpoles depended on the interaction between mass difference and relatedness between dyads. We used biting as a measurement of first aggression because it consistently represented the first aggressive contact in pairs. Based on a mixed effect Cox proportional hazards model, we assessed the risk of first attack when considering relatedness and mass difference between pairs. We detected a significant interaction between relatedness and mass difference, where closely related pairs displayed more immediate aggression when dyad mass differences were large. In other words, siblings bit their counterpart faster when mass differences between pairs were greater (Cox mixed effects, *z *= 2.209, *P* = 0.022, see Table 3). For example, at a large mass difference (>0.15 g between tadpoles) siblings were more than 40% more likely to bite than non-siblings within the first 5 min of a trial. Interestingly, non-siblings demonstrated a seemingly inverted behavioral trend, where dyads with large mass differences had delayed aggression. Half-siblings did not behave significantly differently from either treatment. In trials where biting was exhibited, large tadpoles were most often the first aggressor (*n* = 8/13 for siblings; *n* = 10/13 for half-siblings; *n* = 11/12 for non-siblings).

**Table 3:** Mixed effects Cox proportional hazards model. Time to first aggressive behavior was predicted by the interaction of the mass difference between tadpoles and their relatedness; family is taken into account as a random effect. There is a significant interaction between relatedness and mass, where siblings of similar masses have a shorter latency to aggression than non-siblings. Mass_Diff is the difference in weight between large and small tadpoles

	Latency to first bite			
Predictors	Estimate	CI	*z*	*P*
Half-siblings	−1.27	−2.83–0.30	−1.59	0.113
Siblings	−1.44	−3.12–0.24	−1.68	0.093
Mass_Diff	0.89	−8.65–10.44	0.18	0.854
Half-siblings: Mass_Diff	9.62	−2.14–21.38	1.60	0.109
Siblings: Mass_Diff	16.32	1.80–30.83	2.20	**0.028**

Bold values represent significant values.

### Game theory model

Based on our three formulations (1a–c) we varied the impact of size to model aggression levels of tadpoles with different degrees of relatedness. The version where aggression was both size-dependent and costlier for the smaller tadpoles (Figure 4, third row) appeared most consistent with our empirical data (Figure 2), in that larger tadpoles were consistently predicted to be more aggressive than their smaller counterparts, and overall aggression by large tadpoles decreased with relatedness.

## Discussion

*Dendrobates tinctorius* tadpoles are subject to their parents’ deposition decisions, where males—counter-intuitively—will frequently deposit smaller conspecifics with larger cannibals (Rojas 2014). In this system, the study of how tadpoles interact and manage their aggression is crucial to understanding their father’s unexpected deposition behavior which differs from poison frogs that avoid pools occupied by predatory tadpoles (Schulte et al. 2011). Here, we observed aggression between *D. tinctorius* tadpoles in resource-abundant, low-density conditions. Empirically, we found that aggression is common (Rojas 2014; Fischer et al. 2020), and depends on the interaction between relative size and relatedness between tadpoles. From a theoretical perspective, we found that aggression in this system is probably costlier for smaller tadpoles, as making this assumption yielded predictions that qualitatively matched the empirical observations (formulation 1c, bottom row of panels in Figure 4). Combining empirical and theoretical methods, we found that relatedness and physical attributes interact in shaping overall aggression, latency to aggression, and even activity levels in a context-dependent way.

### Interacting predictors of aggression

In animals where aggression can escalate to cannibalism, the majority of studies focus on the causes that underlie the killing and consumption of conspecifics. This previous work has been primarily done in insects (but see Dugas et al. 2016b for a similar study done with spadefoot toads) and has yielded a variety of results (interaction between relatedness and size: Dobler and Kölliker 2011, relatedness effect only: Bilde and Lubin 2001; Roberts et al. 2003), providing no consistent pattern to extrapolate to cannibalistic vertebrates. In *D. tinctorius*, where there is high offspring investment (i.e., male parental care and low fecundity), we found that large tadpoles (where size is relative between pairs) from sibling dyads were the least aggressive, expressing almost half the amount of aggression compared with large tadpoles from non-sibling dyads (Figure 2). The importance of size differences in predicting aggression was expected: Rojas (2014) established that cannibalism between *D. tinctorius* tadpoles occurs faster with increasingly size-mismatched pairs. In fact, across the animal kingdom, the aggressor in a pair/group is most often the larger individual, which typically faces a smaller risk of injury (Mock et al. 1987; Mayntz and Toft 2006; Ibáñez and Keyl 2010). However, our findings highlight that in this system aggression is not solely mediated by size differences, but that some form of kin discrimination is also at play. To understand the influence of potential kin recognition in modulating aggression, we designed our experiment to include a range of relatedness coefficients between pairs (*r* = 0.5 full-sibling; *r* = 0.25 half-sibling; *r* = 0 non-sibling). Surprisingly, these half-siblings did not differ significantly from either of the other relatedness treatments. Overall, large tadpoles from the half-sibling treatment exhibited similar mean levels of aggression as large tadpoles from the non-sibling treatment, suggesting that if kin recognition does occur it may not function on as fine a scale as for other cannibals (i.e., aversion to cannibalizing cousins in salamanders, Pfennig et al. 1994).

When we compare activity and aggression results, these data reveal a potential mechanism by which tadpoles assess one another. Initially, during the acclimation period we found no differences in activity across treatments (Figure 1A). At this stage, individuals were separated by an opaque physical barrier, therefore removing visual information, but shared the same water, allowing for the transmission of chemical cues. Once the experiment began, the barrier was removed and the tadpoles were allowed to physically interact: here, all large tadpoles within dyads were significantly more active than their smaller counterpart (Figure 1B). Intriguingly, though all large tadpoles across relatedness treatments had similar levels of activity, only non-siblings frequently shifted action into attack. Non-siblings exhibited twice the amount of aggression towards their smaller counterpart than siblings, despite swimming the same amount (see Supp. Fig 2). While we are unsure what cues are being used to discriminate kin in this species, it appears that the visual assessment of conspecifics could play a role in aggressive decisions (the role of vision in activity has also been shown by Kumpulainen et al. in preparation). Overall, recognition amongst larvae is relatively common in amphibians (Waldman 1984; Blaustein and Waldman 1992) and in combination with our latency data (that suggests that initial aggression is shaped by the interaction between relatedness and size differences between pairs), we hypothesize that *D. tinctorius* tadpoles may be using both olfactory or taste cues to discriminate kin (as shown in salamanders, Pfennig et al. 1994 and *Xenopus* sp., Dulcis et al. 2017). Kin discrimination then appears to be used in a context-dependent manner depending on size differences between pairs, which through visual assessment can serve to initiate or escalate aggression. When we consider this from an evolutionary perspective, the context-dependent nature of aggression suggests that the value of kin discrimination is lower in this species; it may be that aggression provides an overall benefit in securing resources or that, in some cases, escalated aggression (and eventual cannibalism) benefits individual survival enough to outweigh the fitness costs of consuming kin.

### The escalation of aggression

The escalation of aggression between individuals is often overlooked or dismissed in systems where cannibalism occurs. These behavioral data can be valuable in understanding opponent assessment and decision-making in cannibals, as there may be unexpected costs paid in terms of energy expenditure and opponent retaliation that are shaping aggressive encounters. Intuitively, one might expect that there would be less aggression between size-mismatched pairs as large size differences may provide a cue to the smaller individual that it is unlikely to win (seen in salamanders: Brunkow and Collins 1998); however, in cases of extreme intraspecific aggression (i.e., the possibility of escalating to cannibalism), aggressive interference models predict aggressive encounters to occur more frequently with increasing size differences (Persson 1985; Polis 1988).

Here, aggressive attacks between pairs were recorded across all relatedness treatments and sizes. Although less common, small tadpoles were sometimes quicker to exhibit aggression than their larger counterparts (Figure 3) and, in some instances, were even more aggressive than large tadpoles (this was observed only in sibling and half-sibling treatments). Latency to attack changed as a function of mass differences between pairs and the magnitude of this change was dependent on relatedness. When pairs were closer in weight, non-siblings attacked faster; in contrast, when mismatched in weight, non-siblings delayed aggression (Figure 3). This trend was inverted for siblings, which were tolerant of a similarly sized counterpart, but were quickly aggressive in pairings with large differences in weight. We speculate that fast “attacking” may serve different functions in different contexts. For example, when performed between mass-mismatched siblings, quick aggression may serve not to initiate cannibalism but to ascertain by taste the first impression of relatedness. While these data may be useful in unraveling potential assessment mechanisms, they should be interpreted with caution as considerable variation occurred throughout non-sibling treatments.

When comparing our empirical data with our inclusive fitness models, we are able to reject several theoretical possibilities for the *D. tinctorius* system. For example, if (adult) reproductive success and aggressiveness costs were independent of tadpole size, smaller tadpoles should compensate for their size disadvantage by being more aggressive (top row of panels in Figure 4). Empirically, we continuously see that small tadpoles are the least aggressive across treatments, suggesting that aggression for small tadpoles is costlier and/or less beneficial. Additionally, if tadpole size strongly predicted adult reproductive success, then above certain size difference smaller tadpoles should let their larger relative win without fighting (middle row of panels in Figure 4). This altruistic behavior of “sacrificing” oneself to a larger relative seemed plausible a priori in light of the observation that, in this system, fathers deposit younger individuals in occupied pools which could function to feed older siblings (Rojas 2014). However, this possibility can be rejected based on our empirical observations, where neither the small nor large tadpoles in pairs fully abandoned their aggressive behaviors towards one another (although we do observe a reduction in aggression in related tadpoles with larger size differences; see Supp. Fig 1 for aggression plotted across mass differences between pairs). When we frame these results in the context of deposition decisions by parents, we could hypothesize that some form of bet-hedging by fathers is occurring when choosing larval nurseries; ultimately, the benefit of a high-quality nursery may be worth the risk of cannibalism when that risk is minimized by being placed with siblings.

## CONCLUSIONS

In this study, we explored aggression under resource-abundant, low-density conditions, which differs from the experimental set-up through which extreme intraspecific aggression is usually reported, such as in response to starvation (Mayntz and Toft 2006; Ibáñez and Keyl 2010; Dobler and Kölliker 2011), pathogens (Pfennig et al. 1991; Wang and Daane 2014), and high population densities (Moksnes 2004). We show here that intraspecific aggression (which may escalate to cannibalism) by *D. tinctorius* is not random, and that the interaction between relative size and relatedness shapes a cannibal’s decision to attack.

We found that large tadpoles from sibling dyads were significantly less aggressive than large tadpoles from non-sibling dyads towards their smaller counterpart, presenting evidence for context-dependent kin discrimination in *D. tinctorius*. These findings are complicated by latency to aggression, which showed unexpected trends based on dyad relatedness, but may be related to the modalities involved in kin recognition. These results set the stage for studies to consider aggression in cannibals in more complex ways, and to better understand the value and purpose of kin discrimination in cannibals.

## Supplementary Material

arac020_suppl_Supplementary_Materials
