# Supplementary material for: Size-dependent aggression towards kin in a cannibalistic species
Source: Behav Ecol. 2022 Mar 25;33(3):582–91. doi: 10.1093/beheco/arac020 (PMC9113263; doi:10.1093/beheco/arac020)

| **Behavior** | **Description** |
| --- | --- |
| Resting | Non-movement under normal, healthy conditions |
| Swimming | Movement of the tadpole’s tail in over two consecutive oscillations without ‘intent’ (see below) with respect to the other tadpole. Sometimes tadpoles contract their muscles or displace by floating up or down the water column; these behaviors did not qualify as swimming in this experiment. |
| Biting | A tadpole attempting or successfully grasping/clutching any body part of another. This behavior was allowed if it was isolated and its duration lasted less than 2 seconds. If the bite was followed by a “hold” by a tadpole that could result in permanent injury or death of the counterpart, the trial was terminated and categorized as ended by a means of “potential lethal attack”. |
| Chasing | A tadpole swimming after its counterpart with intent; “intent” was perceived by the observer as rapid changes in swimming speed and direction of swimming being towards the other tadpole |

Supplementary Table 1. Categorization of behaviour observed during aggression trials between tadpoles.

Supplementary Figure 1. *Differences in aggression across relatedness treatments with respect to weight difference between dyads. Pink dots represent large tadpoles and blue dots represent small tadpoles (sizes relative to dyads); N_Trial_ = 15 for each relatedness level. There was a higher level of aggression by large tadpoles overall, but significantly less aggression by large siblings when compared to non-siblings. Lines fit with GLM smoother (y ~ log(x)) for relative sizes, shaded regions represent 95% confidence intervals around model estimate.*


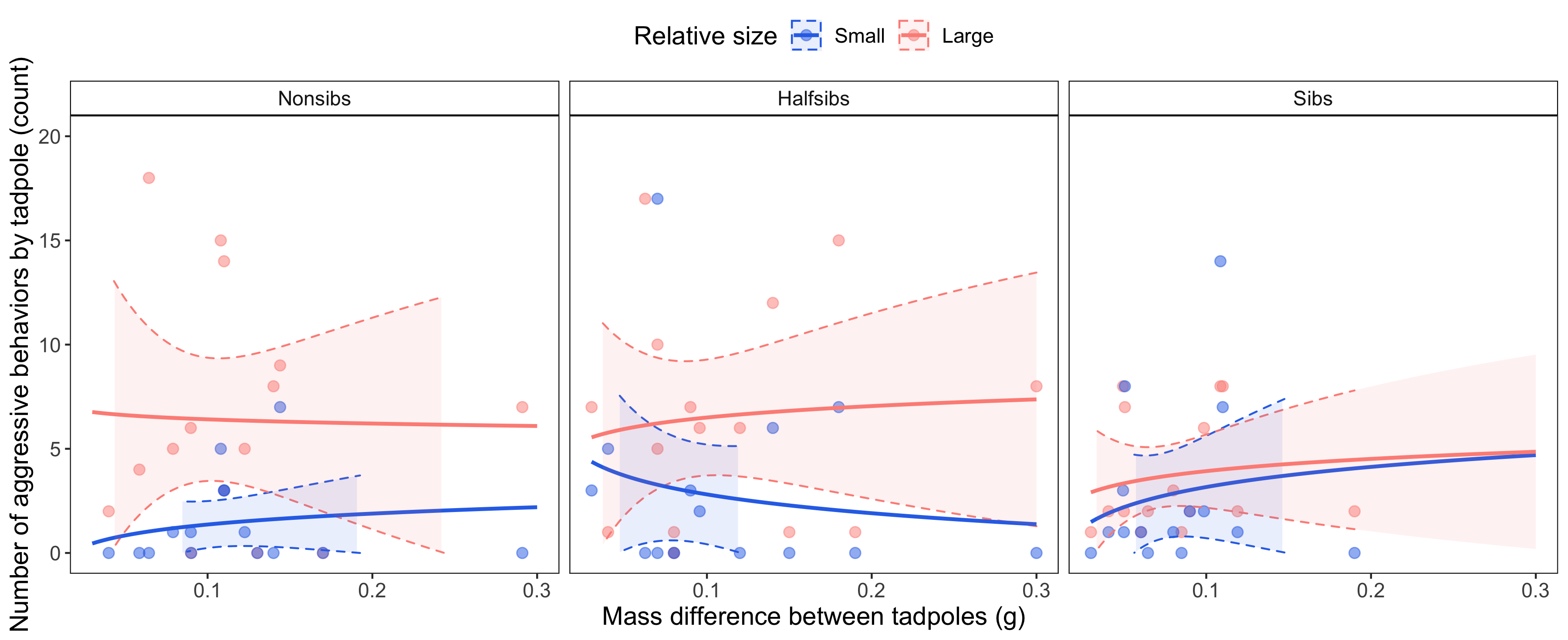


*Supplementary Figure 2. The relationship between experimental activity and tadpole aggression based on relative size. We find for large tadpoles within dyads that activity is strongly related to aggression, especially for non-siblings and half-siblings (pink and red points). Large tadpoles from sibling dyads swim just as much as other treatments, but attack only half as much (Fig 2, 3 main MS). These data provide support for the hypothesis that tadpoles assess their counterpart (swimming, Fig 2. of main text) which informs their overall aggression. Lines fit with a GLM smoother, shaded regions represent 95% confidence intervals.*


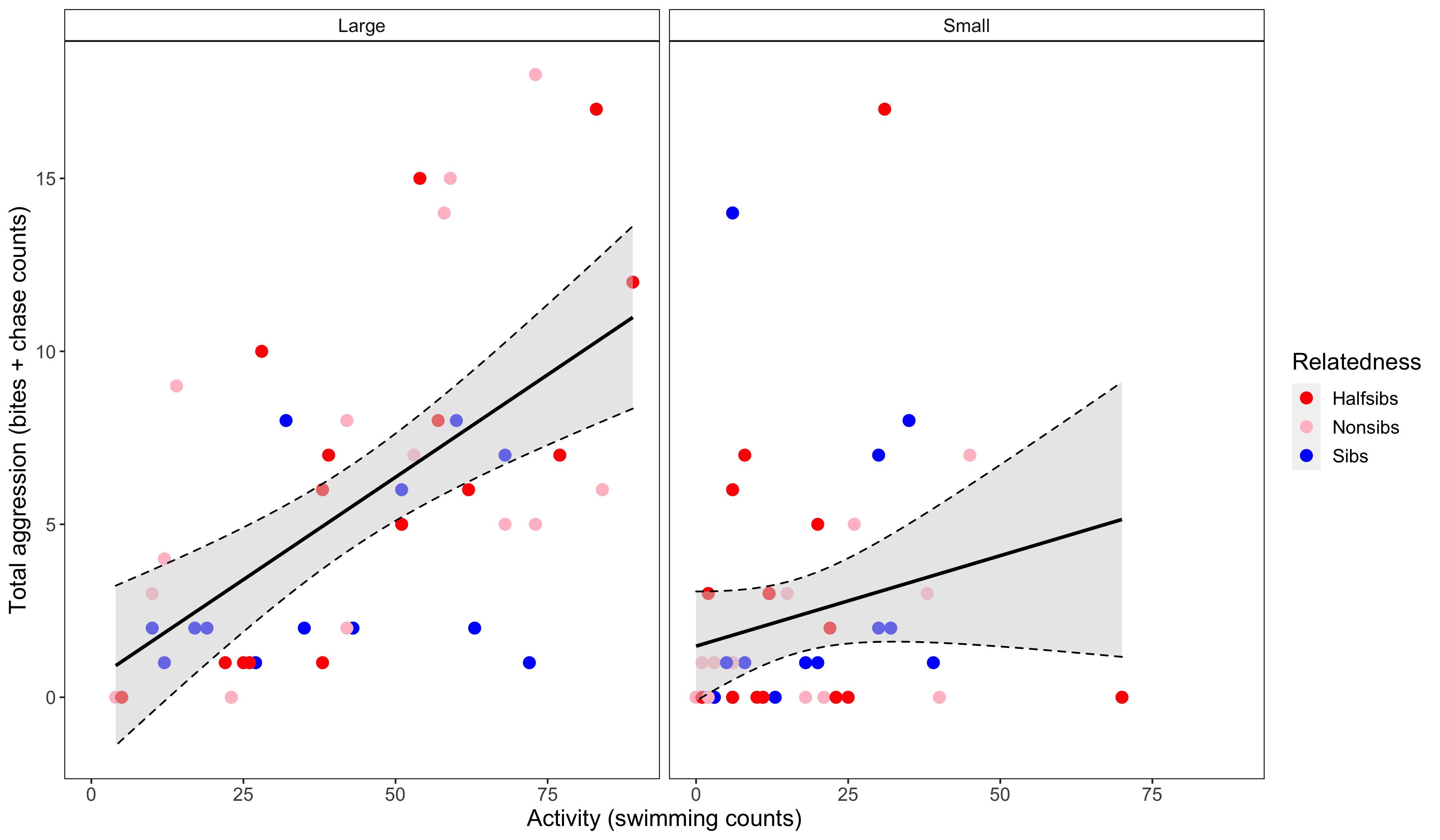

Supplement: arac020_suppl_Supplementary_Materials [file arac020_suppl_supplementary_materials.docx]
